# Brain–Computer Interface (BCI) Applications in Mapping of Epileptic Brain Networks Based on Intracranial-EEG: An Update

**DOI:** 10.3389/fnins.2019.00191

**Published:** 2019-03-27

**Authors:** Rafeed Alkawadri

**Affiliations:** ^1^Human Brain Mapping Laboratory, Department of Neurology, University of Pittsburgh, Pittsburgh, PA, United States; ^2^Yale Human Brain Mapping Program, Yale University, New Haven, CT, United States; ^3^The Department of Neurology, School of Medicine, Yale University, New Haven, CT, United States

**Keywords:** high frequency oscillations, high frequency brain stimulation, single pulse electrical stimulation, BCI, epilepsy surgery, coherence analysis, epileptogenicity index, connectivity index

## Abstract

The main applications of the Brain–Computer Interface (BCI) have been in the domain of rehabilitation, control of prosthetics, and in neuro-feedback. Only a few clinical applications presently exist for the management of drug-resistant epilepsy. Epilepsy surgery can be a life-changing procedure in the subset of millions of patients who are medically intractable. Recording of seizures and localization of the Seizure Onset Zone (SOZ) in the subgroup of “surgical” patients, who require intracranial-EEG (icEEG) evaluations, remain to date the best available surrogate marker of the epileptogenic tissue. icEEG presents certain risks and challenges making it a frontier that will benefit from optimization. Despite the presentation of several novel biomarkers for the localization of epileptic brain regions (HFOs-spikes vs. Spikes for instance), integration of most in practices is not at the prime time as it requires a degree of knowledge about signal and computation. The clinical care remains inspired by the original practices of recording the seizures and expert visual analysis of rhythms at onset. It is becoming increasingly evident, however, that there is more to infer from the large amount of EEG data sampled at rates in the order of less than 1 ms and collected over several days of invasive EEG recordings than commonly done in practice. This opens the door for interesting areas at the intersection of neuroscience, computation, engineering and clinical care. Brain–Computer interface (BCI) has the potential of enabling the processing of a large amount of data in a short period of time and providing insights that are not possible otherwise by human expert readers. Our practices suggest that implementation of BCI and Real-Time processing of EEG data is possible and suitable for most standard clinical applications, in fact, often the performance is comparable to a highly qualified human readers with the advantage of producing the results in real-time reliably and tirelessly. This is of utmost importance in specific environments such as in the operating room (OR) among other applications. In this review, we will present the readers with potential targets for BCI in caring for patients with surgical epilepsy.

Technology alone is not enough–it’s technology married with liberal arts, married with the humanities, that yields the results that make our heart sing.

Steve Jobs

## Current Standards and Recent Advances in Seizure Localization and Intracranial EEG

Intracranial-EEG (icEEG) indicated in the subset of patients with drug-resistant epilepsy (i.e., patients who failed two anti-seizure medications, as mono-therapy or in a combination, composing approximately a one-third of all patients with epilepsy), may present a few challenges:

(i) icEEG is invasive and may present complications, which increase in rate as a function of duration of recording (Fong et al., 2012) (ii) The chances of sustained seizure freedom after epilepsy surgery falls between 30 and 80% (Jehi et al., 2009; Simasathien et al., 2013) depending on the lobe involved suggesting that the current methods of localization are not optimal and approaching epileptogenicity implying zones, while practical, falls short of “ideal” (ii) recording of seizures remain to date the best surrogate marker of the epileptogenic zone which may not be always feasible even after a few weeks of EEG recording (Asano et al., 2009). There are only a few exceptions where the interictal profile, may be adequate for localization of the epileptic tissue in the operating room (OR) such as in focal cortical dysplasia (FCD) (Tripathi et al., 2010).

**FIGURE 1:**
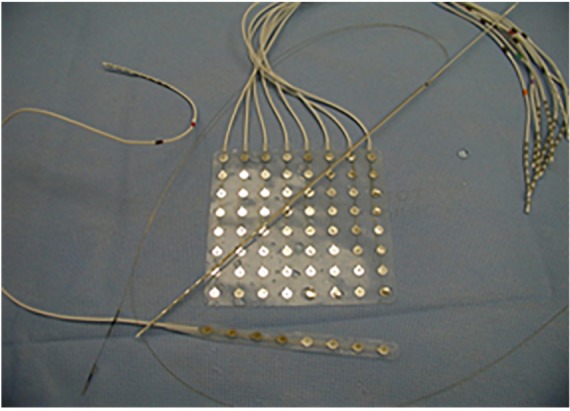
Different type of electrodes currently employed in practices. The depth electrodes and stereo-EEG commonly employed in Europe especially in France, and more recently in the United States. Whereas subdural electrodes constituted the mainstay of evaluations in the United States until the last few years.

A standard single electrode (Figure 1) provides an estimate of the field potential of the summation of excitatory and inhibitory post-synaptic evoked potentials roughly from 100 million to 1 billion of neurons. Electrocorticography (ECOG) has the advantage of proximity to the source of electrical activity only separated by highly conductive media and low impedances. Using simultaneous scalp and intracranial recording, cortical spike sources having an area of 10 cm^2^ or more commonly resulted in scalp-recordable EEG spikes (Tao et al., 2007). ECOG is less susceptible to artifact and provides higher signal-to-noise ratio. Additionally, depth electrodes allow exploring mesial brain structures and deeply seated foci not accessible otherwise.

Indication: The traditional goal of epilepsy surgery is to disconnect the epileptogenic zone which is the area of the cortex indispensable for seizure generation, and which resection leads to seizure freedom (Table 1). The decision about implantation is discussed during a multi-disciplinary surgical conference attended by neurosurgeons, neurologists, neuropsychologists, radiologists, trainees, and nurses among others. The typical indications include:

**Table 1:** Summary of Cortical Zones and their assessment with clinical tools.

Cortical zone	Methods of assessment
Ictal onset zone	EEG, ictal SPECT
Irritative zone	EEG, Magnetoencephalography, functional MRI triggered by EEG
Symptomatogenic zone	History and semiology
Epileptogenic lesion	MRI
Eloquent cortex	Cortical stimulation, functional MRI, evoked potentials, Magnetoencephalography
Functional deficit zone	Physical examination, neuropsychiatric testing, EEG, PET, SPECT, MRS
Epileptogenic zone	None (theoretical construct)

• Discordant non-invasive pre-surgical work-up
• MRI-negative neocortical epilepsy and select cases of mesial temporal epilepsy
• MRI-lesional cases if:
  ◦ Adjacent to eloquent cortex
  ◦ Detailed language or functional mapping needed
  ◦ Plan to maximally define the epileptic zone for completeness of resection such as in focal cortical dysplasia (FCD)
  ◦ Dual pathology or multi-focality (i.e., tuberous sclerosis)
  ◦ If discordance with EEG data (i.e., scalp EEG is non-localizable).

The standard approach is to record seizures in the epilepsy monitoring unit.

### Intra-Operative ECOG

There is somewhat conflicting evidence to support precision of pre- and post-resection ECOG for localization of the epileptic focus, owing to the heterogeneity and the retrospective non-randomized or non-controlled designs in the available studies and the multiple clinical variables to control. That is, some studies correlated resection of spikes with seizure freedom (Palmini et al., 1995; Bautista et al., 1999; Sugano et al., 2007; Stefan et al., 2008; Tripathi et al., 2010) but not others (Cascino et al., 1995; Kanazawa et al., 1996; Tran et al., 1997). Several studies have suggested that residual spikes in the final post-ECoG predict poor surgical outcome (Wennberg et al., 1998; Oliveira et al., 2006), but this is again contradicted by others. Recording in the intra-operative settings may be adequate in

• Select-cases in children especially younger ones
• Lesions with concordant non-invasive evaluations in focal cortical dysplasia
• As an adjunct in multiple-subpial-transections (MSTs)
• Adjunct during intra-operative monitoring and mapping of eloquent cortex
• As an adjunct in placement of Responsive Neuro-Stimulation (RNS) electrodes.

Limitations of intra-operative ECOG: Seizure onset almost always not recorded in the operating room. Chemical induction fell out of trend. There is a tunnel-vision related to the limited spatial sampling. Thus, successful localization must be guided by a strong clinical hypothesis. Anesthesia may limit the analysis of epileptic activity. The ideal agents for intra-operative recording are those with minimal effect on baseline spike frequency. Inhaled agents tend to suppress background EEG activity, with reports of enflurane (Flemming et al., 1980) and sevoflurane (Dahaba et al., 2013) exhibiting activating effect. Synthetic opiates such as remifentanil and alfentanil may increase the yield of recording epileptiform activity (McGuire et al., 2003). The latter may induce non-habitual seizures from healthy brain regions. A few studies have shown that dexmedetomidine has no or little activating effect on epileptiform activity (Chaitanya et al., 2015). Propofol, barbiturates, and benzodiazepines increase EEG background beta-sigma frequency-power and may obscure epileptiform discharges (Dahaba et al., 2013; Nishida et al., 2016; Bayram et al., 2018a).

### Surgical Outcomes and Safety

Over the past decade, there has been a plethora of literature reporting on long-term outcomes following epilepsy surgery with chances of long-term and sustained seizure freedom ranging from 30% in frontal lobe epilepsy and up to 80% in lesional mesial temporal lobe epilepsy. This outcome compares favorably to a 5%-per-year chance of seizure freedom using anti-seizure medications alone in medically intractable cases (Callaghan et al., 2011). Duration of implantation correlates with the histopathological changes such as micro-hemorrhages and inflammatory response (Herman et al., 2017). Commonly, electrodes are removed within 3 weeks following implantation. There has been a steady decrease in risk of complications with advances in surgical techniques (Yuan et al., 2012; Arya et al., 2013; Herman et al., 2017).

For the aforementioned, any future electrophysiological markers ideally would emphasize both efficiency and reliability in classification of brain tissue. Studies of markers that are more specific of localization of the epileptogenic brain regions in the interictal phase are always welcome and still badly needed preferably as part of large, multi-center consortia.

## Recent Advances in Brain–Computer Interface (BCI)

**FIGURE 2:**
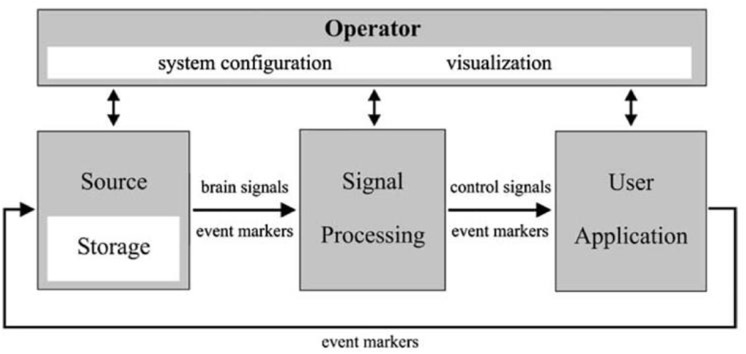
A schematic showing a universal design for BCI systems. Adapted from BCI2000 IEEE TRANSACTIONS ON BIOMEDICAL ENGINEERING, VOL. 51, NO. 6, JUNE 2004.

The brain–computer interface (BCI) (Figure 2) is a device that reads voluntary changes in brain activity then translates these signals into a message or computational command in real-time. It is a method to communicate with the brain, that does not depend on the brain’s normal output pathways. Current BCI’s record electrophysiological signals using non-invasive or invasive methods. These BCIs can provide a much more detailed picture of the brain activity, which can facilitate prosthetic applications or surgery for epilepsy and tumor removal. The emphasis in this article is to describe possible applications to enhance care for patients affected by drug-resistant epilepsy via invasive sensors and electrophysiology. For other applications and for BCI engineering aspects please refer to other sections.

The applications of BCI are more relevant nowadays given recent advances in the sampling of icEEG and in computational power. The amount of information encoded within the icEEG that is untapped into on a regular basis is enormous. It appears that interacting with live-streaming electrophysiological data, sampled at high-frequency, and processed in real-time will be the future, by natural, or artificial for this matter, evolution.

In general, the critical components of BCI are:

1. Sensors: In this case is an intracranial icEEG electrode placed at the surface of the brain.
2. Translation for communication: Programming language and commands.
3. Real-time acquisition and processing of EEG signal. This includes EEG amplifiers enabling access to data as they are recorded.

Different methods of interfacing are available:

1. Many of the commercially available EEG amplifiers may provide Software Development Kits (SDKs) to enable an interface with EEG signal as it is acquired. Skills in programming and software development are required.
2. EEG amplifiers designed for interfacing with widely used languages in signal and image processing such as MATLAB^®^, Simulink^®^, and python^®^. This approach is commonly employed for development, in academia, and for research.
3. One or multi-purpose integrated end-to-end hardware and software. The best consumer experience (patient and practitioner in this case), as learned from industry, come from the “whole widgets” kind of products with the software carefully tailored to the hardware and vice versa. Commercializing the BCI applications in the field of epilepsy surgery will likely follow this path.

A remarkable amount of funds has been raised to support research in BCI and its applications in the private sector over the past few years. It is only logical if parallel strides are taking place in the epilepsy world, so that the community and researchers with a specific interest in management of drug-resistant epilepsy, could tap into and benefit from the growing popular interest.

## Current BCI Applications in Drug-Resistant Epilepsy

This is an area at the intersection of multiple disciplines of science and has yet to be integrated in clinical practices in the broader sense. Ongoing parallel research is in progress. Developing algorithms tailored for clinical use, beyond abstract research-statistics, and validated by surgical outcomes continue to be needed; one challenge is that parametric statistics are often not clinically compelling, hence, expert-driven non-parametric evaluation of results will most likely benefit the clinical applications (Maris and Oostenveld, 2007). Multi-center efforts are required in order to increase the number of cases and hence the statistical power of the findings. Among other methods, the iEEG.org portal provides a potential seed for collaboration and data sharing.

**FIGURE 3:**
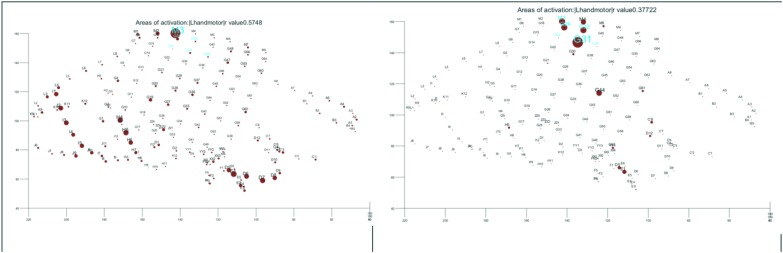
Function: **(Left)** hand motor according to commonly employed parametric methods of analysis of task-related gamma activation. **(Right)** Improving on the results by custom made algorithm. The results of direct electrical cortical stimulation are highlighted in cyan both figures for references. All shown in the electrode space. The size of the dot is proportional with the strength of task-related activation.

**FIGURE 4:**
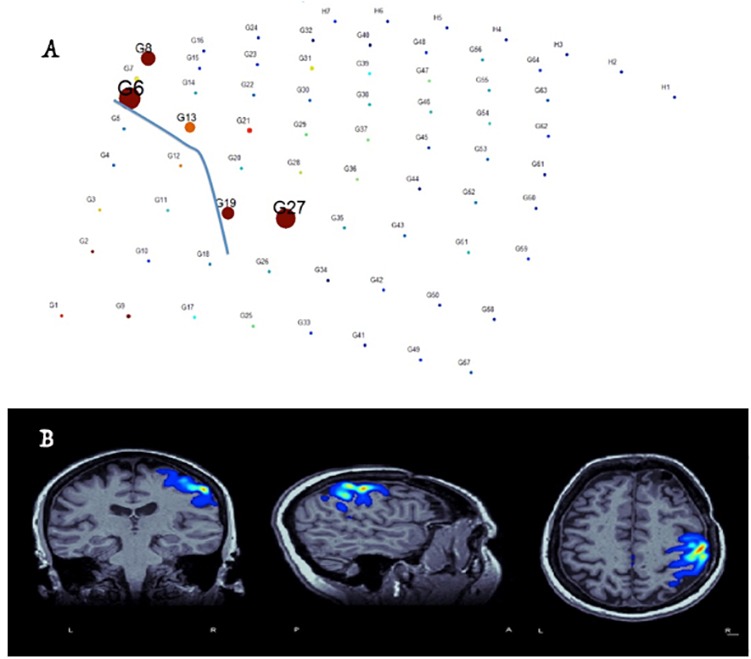
Identification of hand-motor area and the central sulcus (blue-line in electrode plain) and co-registration of the results with brain MRI in real-time based on free-running ECOG and custom-made software (Alkawadri et al., 2015). **(A)** Size of circles corresponds to the value M specific to the anterior lip of the central sulcus – whereas the color represents local field normalized power in the electrode space. **(B)** Co-registration of M values in the MRI space and proper thresholding to demonstrate the localization of the hand area.

Some of the known BCI applications in caring for patients with drug-resistant epilepsy include:

* Real-time localization of the language centers especially in patients in whom current gold standards are not applicable. The most practical use at the present point is to make mapping by electrical cortical stimulation (ECS) more efficient, by supplementing the planning process. It is our experience, however, (Figure 3) to encounter false-detections in the clinical-sense in the 1. Occipital and junctional regions 2. Frontal attention network and 3. The epileptic brain regions. In fact, the issue of spatial sampling is a pertinent one in any research involving icEEG, as generally speaking there is a consistent bias toward sampling from epileptic brain regions, and this fact should be incorporated into the interpretation of available literature reporting on predictive values of markers of function or epilepsy.
  It is an active area of research to optimize detections within functional brain regions that are most important for the surgical decision and to exclude less relevant ones (i.e., increase the clinical specificity). This will require employing more steps than simple parametric and energy-based detection of gamma activity (Alkawadri and Gaspard, 2018). Elaborating on current non-parametric methods and expert validated outcomes will likely benefit practices. For instance, we have been able to optimize methods to localize the hand motor area (Alkawadri, 2017; Alkawadri et al., 2015), and the entire sensorimotor strip successfully during sleep (Figure 4).
* Responsive Neuro-Stimulation (RNS) for seizure detection and electrical stimulation for modulation and management of drug-resistant epilepsy.
* Near-Instantaneous classification of perceptual states from cortical surface recordings.
* Real-time seizure detection.

In the next few sections, we will present areas and markers in surgical epilepsy with BCI potential.

### Epileptiform Discharges

Interictal EEG spikes are known to be categorically correlated with the presence of epilepsy. However, interictal discharges can be seen also in areas other than SOZ and tissues distant from the epileptic tissue (Jasper et al., 1961). Jasper’s early work had led to the conclusion that not all spikes are equal and that there are ones that are more localizing of the epileptic region than others.

**FIGURE 5:**
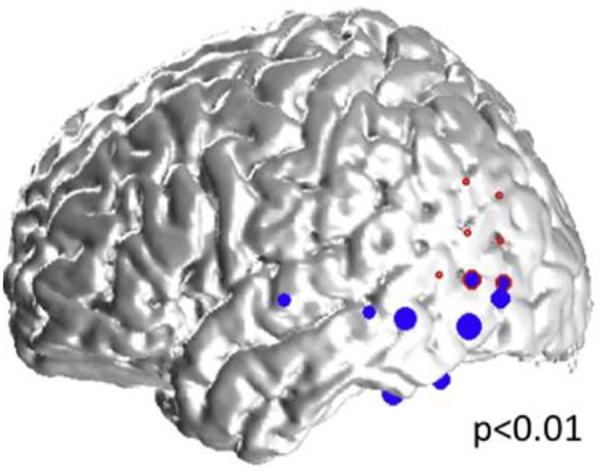
Representative case: 34-year old woman with left neocortical temporo-parietal epilepsy. The size of the blue dots represents the “spikiness” of the automatic-detected spikes which are located in the left inferior and lateral temporal lobes, as well as in the left inferior parietal lobe, and overlap partly with, and within the vicinity of the seizure onset zone (red circles).

The agreement between the seizure zone and the irritative zone, however, is estimated at approximately 56% based on a surgical series and more so in focal cortical dysplasia FCD ∼75% (Bartolomei et al., 2016). The prominent spikes tend to arise mostly from contacts located in the close vicinity of the seizure onset area rather than from within it. We found that the most sharply looking ones are those in the vicinity of the seizure onset zone rather than precisely within it (Gaspard et al., 2017) (Figure 5). More recently, there has been a suggestion that high-frequency oscillations co-occurring with spikes are highly specific for the seizure-onset zone (Wang et al., 2013). In fact, co-occurrence increases the specificity for both (Ren et al., 2015). Due to Gibbs phenomenon, fine-tuning of reliable detectors of spike-HFOs, especially those that are based on non-sine methods, such as wavelet spectral analysis or Hilbert transformation and power ratios in different bands, appear more efficient than standard energy-based ones (Birot et al., 2013).

### Intraoperative Spike Monitoring

Extra-operative video-EEG monitoring and recording of the seizure onset remain to date the best surrogate marker of the epileptogenic zone. Until a reliable interictal biomarker is available for the clinical decision making, there remain situations where the intra-operative monitoring is desired. “Spike chasing” and “tailored resections” may not lead to desired outcomes if not supported by a valid hypothesis. Randomized controlled trials are still needed as there is a somewhat a lack of strong evidence on the best use of it. Some studies have suggested that residual spikes in the final post-ECOG predict poor surgical outcomes, but this was contraindicated by other studies. A primary concern is that surgical manipulation of the cortex may agitate the tissue, evoking spikes in the resection margin, which are not correlated with the seizure outcome. Spike-HFOs may present somewhat a more reliable and specific interictal biomarker for the epileptogenic brain region in that settings.

Until these studies are completed, the concept of intraoperative localization of the epileptic focus will remain an active area for fine-tuning. The clinical implementation of HFOs will be hampered by the existing gaps of knowledge such as the need to discriminate between physiological and pathological HFOs and the requisite for reliable computational detection methods that address existing concerns. The first randomized, controlled, clinical trial (The HFO Trial) to evaluate our hypothesis that use of HFOs intraoperatively can improve outcome is underway. Furthermore, it is important to fill a critical gap of the effect of anesthesia and the ideal anesthetic regimen for intraoperative ECOG monitoring. There is no consensus in regard to the ideal anesthesia regimen for intraoperative monitoring. Existing studies often do not employ common gold standard for localization, and the concept of pharmacological spike activation is challengeable. In our review of literature, of the five studies only provided specifics on site of resection and correlation with surgical outcomes out of 23 stduies reporting on spike activation of a total altogether 54 studies that met inclusion criteria (Bayram et al., 2018b).

### High-Frequency Oscillations (HFOs) and Very High-Frequency Oscillations (VHFOs) – See Also the Previous Section

**FIGURE 6:**
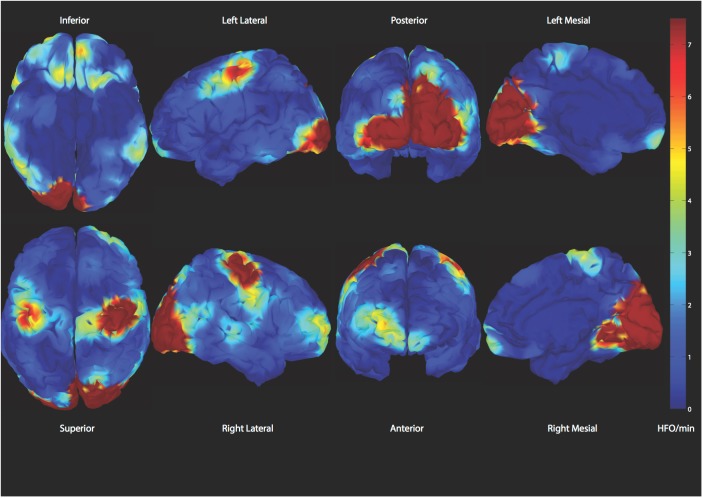
Demonstration of spatial distribution of physiologic high frequency oscillations. A reliable classifier to distinguish those from epileptic ones is desired (Alkawadri et al., 2014).

**FIGURE 7:**
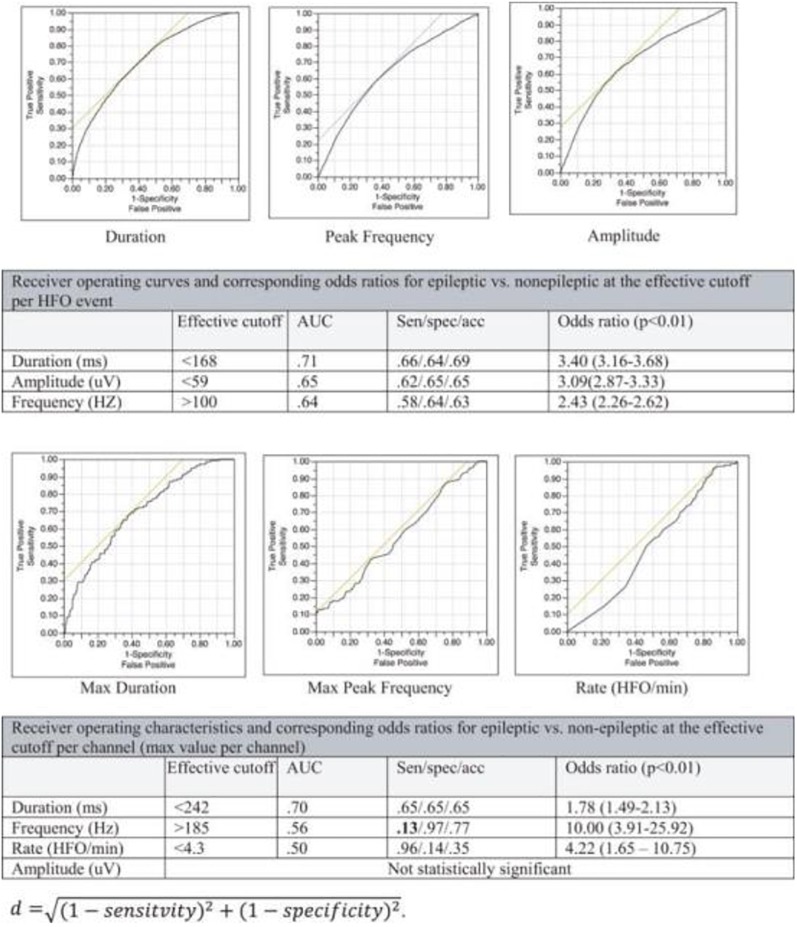
To-date no single EEG feature can reliably distinguish epileptic from non-epileptic HFOs.

There has been a growing interest in the utility of interictal high-frequency oscillations HFOs (80–500 Hz, classically) for localization of the epileptic focus (Bragin et al., 1999; Jacobs et al., 2008, 2009a,b). Several challenges arose as similar oscillations have been associated with specific tasks or occur naturally during sleep (Figure 6), and no known signal parameter can reliably distinguish between physiologic and epileptic subtypes in a given individual (Alkawadri et al., 2014) (Figure 7). Hence, interictal HFOs, although useful, are not highly specific and do not replace current standards. Ironically, it appears that to-date, the most effective factor that increases the specificity of HFOs for detection of epileptogenicity is their co-occurrence with other markers, i.e., spikes. One common denominator among studies reporting on HFOs is that the analysis often performed at the group level and case-wise results are not always presented. The latter perhaps is more relevant for the decision-making in clinical practice. Furthermore, the approach of assessing surgical resections and relatively short-term seizure-free outcomes while commonly employed, has inherent limitations. This holds true especially in the face of the concept of ongoing epileptogenesis even after seemingly successful resections (Najm et al., 2013; Simasathien et al., 2013).

Very-high-frequency oscillations (VHFOs), i.e., Oscillations 500–2000 Hz or above may be more specific for localization of the epileptogenic region (Usui et al., 2015; Brazdil et al., 2017). The frequency exceeds the firing-rate of individual single neurons and likely represent a rhythm generated by in- and out- of phase action potentials of neuron clusters.

**FIGURE 8:**
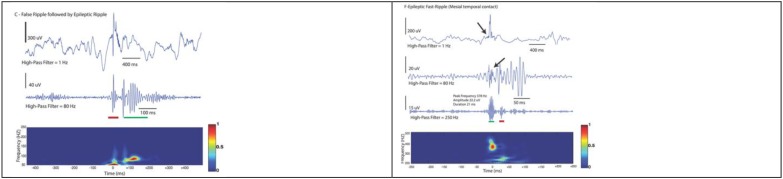
Examples demonstrating a false detection in red (not discerned on raw data and does not occupy a blob in the Morley-Wavelet based spectral window), and true ripple marked by a green line. The spectral analysis is even more important in analysis of fast ripples (lower).

Some of the variable results with HFOs and VHFOs in the existing literature stem from; the extent of spatial sampling (Figure 8); methods undertaken to exclude detections with filtering artifact; the review montage; methods implemented in detection and analysis and effort made to exclude filter-related false detections; areas sampled; size of contacts; time of study and relation to tasks/meds/seizures. Some have employed a battery-powered amplifier system to eliminate the noise of alternating current cycles. Others have subtracted the averaged signal in the white matter from all signals, providing thus additional noise reduction and an optimal reference in theory. It is important to investigate the occurrence of VHFOs outside the epileptic regions, to avoid bias and inflation of positive predictive values resulting from the natural inclination toward sampling epileptic brain regions. The extent of spatial sampling at Yale, has led to detection of ripples and fast ripples consistently at a significantly higher rate outside the epileptic network.

In summary, the ability to sample EEG at high frequencies is perhaps the most suitable tool, in theory, to tap into neuronal communications. Current data seems to suggest, that shorter HFOs bursts, higher-peak-frequencies, and higher entropy in HFOs bands are more suggestive of pathology. No single feature, however, can reliably classify the groups other than perhaps co-occurrence with epileptiform spikes. Presently employing a real-time detection of spike-HFOs and addressing the issue of false detections are of interest. Until then, it appears we are arriving at the time of integrating real-time detection of HFOs/spike-HFOs in clinical practice. Clinician-friendly and commercially available automatic real-time detection algorithms (highly specific) are needed as we continue to advance knowledge on that end.

### Brain Connectivity

Functional connectivity is defined as the study of temporal correlations between spatially distinct neurophysiological events. There are several conceptual distinctions between the different functional connectivity measures: They either reveal directed or undirected, linear or non-linear connections in the time or frequency domains. The calculation is either amplitude or phase-based, and the measure can be bivariate or multivariate. Standard coherence is the equivalent of correlation within a specific band. The temporal resolution of EEG presents a unique modality for analysis of different connectivity and association indices beyond the uni-dimensional correlation coefficient, which is the practical choice in slow fluctuating signals such as those encountered in functional imaging.

The connectivity measures could be conceptually subdivided into four subgroups (van Mierlo et al., 2014):

1. Correlation and coherence: Pearson correlation coefficient, and loosely its equivalent when applied on specific frequency bands. A variant of this measure is the cross-correlation that investigates the correlation between two time-series that are shifted in time concerning each other. The phase of the coherence can be used to infer the directionality. The temporal resolution of EEG presents a unique modality for analysis of different connectivity and association measures beyond the uni-dimensional correlation coefficient, which is the practical choice in a slow fluctuating signal such as the one encountered in functional imaging.
2. Instead of investigating the relationship between the amplitudes of the signals, one could also examine how the phases of the considered signals are coupled, the so-called phase synchronization measures. The most commonly used measures are the phase-locking value and phase-lag index.
3. Information-theory-based, with the most frequently employed is mutual-information and the transfer-entropy which enable investigating non-linear relations.
4. The fourth category of functional connectivity measures is based on the concept of Granger causality for which Clive Granger received a Nobel-prize when invented to be applied in Economics. One time-series is said to Granger-cause the second one if the inclusion of the past values of the first into the modeling of the second significantly reduces the variance of the modeling error. Most of the Granger causality measures are constructed based on an autoregressive (AR) model, in which the present samples of the signals are predicted using a linear combination of the past samples. From the coefficients of the AR model many measures can be derived: The Granger-causality index the directed coherence, the directed transfer function and the partial directed coherence.

None of these methods are perfect, and one should employ depending on the questions, for instance, whether directionality or non-linearity are of interest. Studies have suggested that there is an increase in synchronization in the inter-ictal phase within the resection bed (Avoli, 2014). Recently, it has been shown that high-frequency Granger causality before the actual seizure onset and higher values correlated highly with contacts at seizure onset (Rummel et al., 2015; Park and Madsen, 2018). Also, interictal connectivity within temporal lobe showed more loose patterns as a function of the duration of epilepsy before the surgical evaluation (Englot et al., 2015). Most EEG-based connectivity techniques are research-based, but many will be potentially useful for evaluation of cerebral abnormalities. Further studies to correlate connectivity findings with seizure localization and functional mapping results are still desired and in concept will be a suitable application for BCI.

Some technical issues that should be paid attention to:

1. The quality of recording and montage of review are of particular significance in the setting. For instance, a slightly contaminated reference may result in a false inflation of direct correlation or coherence-based values (Arunkumar et al., 2012).
2. Studies are lacking to correlate spontaneous ECOG-based connectivity measures and other measures of functional connectivity such as fMRI or anatomical connectivity such as Diffusion Tensor Imaging (DTI). We did not find a meaningful correlation between coherence in different frequency bands and cortico-cortical evoked potentials (unpublished work). We hypothesize that this is because stimulation activates complex poly-synaptic networks at a distance, whereas spontaneous connectivity measures identify local networks.

#### The Connectivity Index (CI) as New Measure to Grade Epileptogenicity Based on Single-Pulse Electrical Stimulation (SPES)

Victor Horsley used faradic electrical stimulation to confirm the localization of the epileptic focus in one of John Hughlings Jackson’s patients who underwent resection of an epileptic focus in 1886 (Vilensky and Gilman, 2002). Harvey Cushing used this technique in 1909 to define the sensorimotor cortex surrounding a tumor and to confirm the localization of epileptic seizures that manifested with sensory auras (Feindel et al., 2009). Following the advances in EEG acquisition after 1929 and the standardization of the use of electrical stimulation in brain mapping by Wilder Penfield, induction of seizures through electrical stimulation fell out of favor in North America (Isitan et al., 2018). In Europe, and particularly in France, electrical stimulation continued to be used for seizure induction with variable reports of reliability and specificity for localization of epileptic brain regions (Kovac et al., 2016). Controlled studies on stimulation parameters, efficacy, and specificity of seizure induction are methodologically challenging due to the difficulty in controlling for several covariates. However, our experience aligns with previous reports that suggest seizures produced by 50-Hz stimulation are not specific for localization of the epileptic focus though perhaps more sensitive than 1-Hz stimulation. There is a school that suggests seizures induced by high-frequency stimulation if similar to habitual seizures, i.e., electro-clinical syndrome may be more specific. Our experience in extra-temporal epilepsy aligns well with the limited reports emphasizing the specificity of seizures and auras induced by low-frequency single pulse stimulation in temporal lobe epilepsy.

Recently, we have shown that a new metric we labeled the connectivity index (Alkawadri et al., 2013) which is based on the normalized number of averaged evoked responses to single pulse electrical stimulation weighted by the normalized distance at which the responses recorded at.

$$CI=\frac{n.\overset{¯}{d}}{N.\overset{¯}{D}}$$

C*i* Connectivity index, n, number of contacts with evoked responses, N total number of contacts, d, D average Euclidian distance of contacts with evoked responses and all contacts from site of stimulation, respectively. This measure accentuates responses recorded at different sites. Also, it may bypass some limitations related to the sampling bias (i.e., epileptic areas are more sampled than non-epileptic brain regions).

**FIGURE 9:**
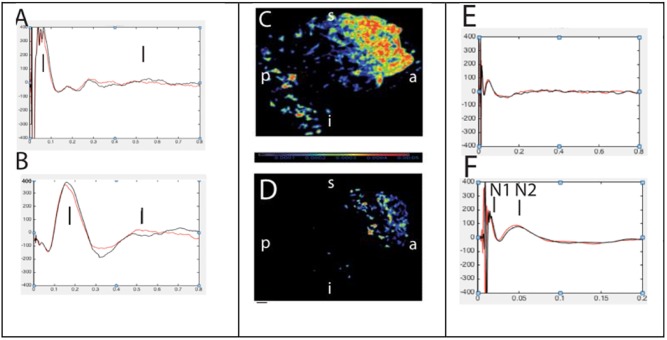
Abnormal (left, **A,B**) and normal evoked responses (right, **E,F**) to single pulse electrical stimulation. Panels **(C,D)** represents source localization of late and slow responses after stimulation of epileptic and non-epileptic orbitofrontal brain regions in two patients, respectively (Alkawadri et al., 2015).

We analyzed responses in thirty-nine stimulation sessions in 19 patients. Stimulation of the epileptic contacts generated reproducible responses at significantly higher rates than the control sites (medians of normalized number of contacts 0.74 vs. 0.32, *p* = 0.0007). These differences were even stronger when normalized to average distance of recorded responses from the stimulation site (medians of normalized values 0.71 vs. 0.15 *p* = 0.0003) (Figure 9). The evoked responses after stimulation of the epileptic contacts were seen at further distance from the site of stimulation (medians of normalized distances 0.93 vs. 0.58, *p* = 0.0004, median absolute values: 58 mm vs. 44 mm). It was 2.2 times more likely to record an evoked response from the seizure onset zone than other contacts after stimulation of a remote-control site. Habitual partial seizures or auras were triggered in 26% of the patients and 33% of the seizure onset contacts (median stimulation intensity 3.5 mA), but in none of the control or within network contacts. Stimulation of control sites in multifocal or poor surgical outcome cases tended to exhibit higher number of evoked responses at distant sites compared to the localizable onsets or good surgical outcome (median number of contacts normalized to total number of contacts and average distance 0.5 vs. 0.12, *p* = 0.06). Stimulation of epileptic contact generated responses with longer latencies (medians 48 vs. 38 ms, *p* < 0.0001), and longer duration (medians 73 vs. 62 ms, *p* < 0.0001). There was a correlation between the current intensity and normalized number of evoked responses (*r* = +0.50, *p* < 0.01) but not with distance (*r* = +0.1, *p* < 0.64), suggesting perhaps that stimulation at lower currents may possibly help in identifying distant nodes within the epileptic network and help differentiating between epileptic and non-epileptic sites. Furthermore, we demonstrated that it is possible to co-register volumes based on abnormal responses to single-pulse stimulation with patient’s MRI for reliable visualization.

### The Epileptogenicity Index (EI), and Other Seizure-Related Metrics

Quantitative seizure analysis is of interest in clinical practice. An expert review remains to-date the mainstay of analysis. Low-frequency high-amplitude repetitive spiking (LFRS) is the most frequently reported pattern in mesial temporal lobe epilepsy and seems to correlate with degree of volume loss. In neocortical epilepsy, focal low voltage fast activity is the most localizing rhythms and it appears that the slower and the more wide-spread the rhythms are, the more likely that site of onset is not sampled, or alternatively this may be viewed as a sign of a complex epileptic networks (Singh et al., 2015). Some authors employed non-parametric and parametric methods for seizure localization and incorporated time to involvement (Bartolomei et al., 2008; David et al., 2011). In our practice, in the majority of the seizures that are poorly localized by conventional clinical analysis, the quantitative EEG analysis identified strongly overlapping networks.

**FIGURE 10:**
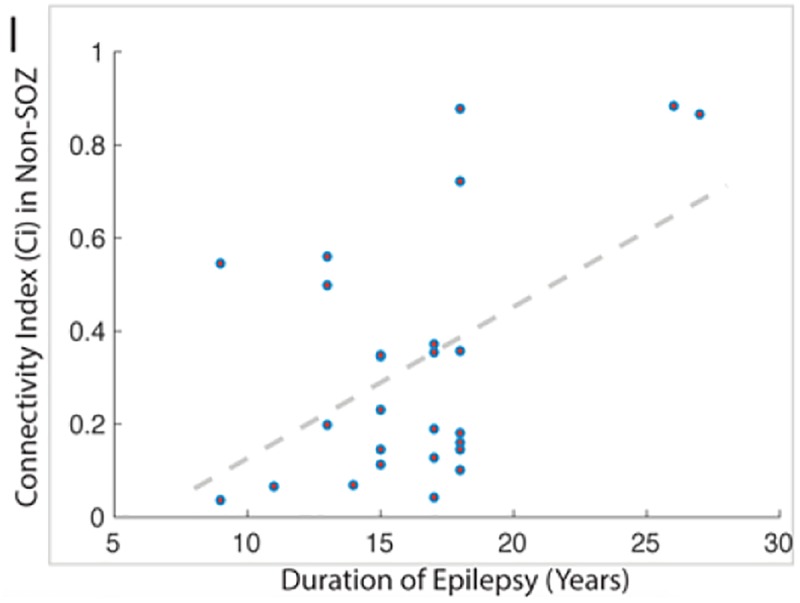
This figure demonstrates the strong correlation between the duration of epilepsy and the degree of epileptogenicity from non-SOZ tissue as graded by the connectivity index.

**FIGURE 11:**
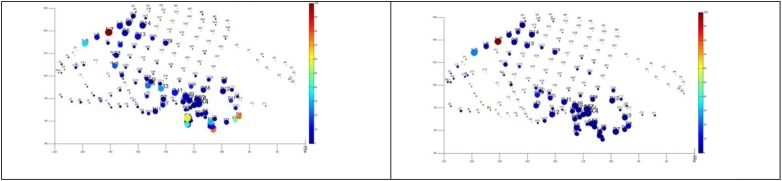
Quantitative analysis based on cumulative ictal high frequency oscillations in two difficult-to-localize seizures. The size represents the length of electrode’s involvement in ictal HFOs, and the color represents normalized cumulative power up to 20 s after seizure onset. Note the strong spatial overlap and that these seizures were interpreted differently by the clinical team as temporal (above), and fronto-parietal (below).

Other benefits of quantification of seizure onset:

1. Evidence showing a correlation between duration of epilepsy and non-SOZ contacts (Bartolomei et al., 2008) compilable with other reports and our observations (Figure 10).
2. Limited evidence suggesting that resection of ictal high-frequency oscillations phsae-locked to lower frequencies/spikes correlate with better surgical outcomes (Weiss et al., 2013).
3. Interestingly, our practice has led us that in difficult to localize cases and even those seizures that are classified of seizure analysis, quantitative analysis of ictal rhythms tends to show somewhat more stable networks (Figure 11).

### Provoked Seizures and Seizure Detection

As presented above, the implementation of electrical stimulation in awake craniotomies predate the discovery of EEG in 1929. As recently reviewed by Kovac et al. (2016), there has been several studies published on the subject and almost all agree that seizures induced by 50-Hz stimulation are not specific for the localization – though more sensitive for induction than 1 Hz. On the other hand, there is a suggestion that seizures recorded with 1-Hz stimulation may be specific for the SOZ, but the technique is less powerful for seizure induction especially outside the medial temporal lobe structures. Seizures can be induced by ECS but there is controversy regarding the utility of ECS induced seizures in defining the epileptogenic zone and hence practice varies considerably between centers. We reviewed the Yale experience with seizures included by electrical cortical stimulation in 24 patients undergoing intracranial EEG evaluation Seizures Provoked by Low Frequency Stimulation. Habitual partial seizures or auras were triggered in 27% of the patients and 35% of all seizure onset contacts that were stimulated with 1 Hz stimulation (median stimulation intensity 4 mA, range 0–59 s from onset of electrical stimulation), but in none of the control or IZ contacts. Only habitual seizures and auras were recorded. None of the evoked auras led to generalized seizures. All but one was focal with retained awareness. There are no non-habitual seizures recorded by 1-Hz stimulation. In relation to BCI, the issue of auto-seizure detection and prediction in real-time becomes of interest. Seizure detection is of interest and has different clinical applications whether based on intracranial EEG, scalp EEG, or other markers. Different seizure detection algorithms exist, most of which achieve sensitivities in the order of 60%-> 90% and false detection rate of <1 seizure – many seizures per hour. It appears that methods based on trained support-vector-machine-learning and artificial neural networks are the ones that achieve the highest performance. As a general rule, the more sensitive a method is the more computationally simple (Ramgopal et al., 2014).

## Future Research and Directions

A quick look at a recent submission to the annual BCI society award and research trends available from the BCI community annual conference shows:

1. There is a steady increasing trend in BCI research with emphasis on epilepsy and movement disorders.
2. This constituted, however, only 3.8% of the projects submitted. These numbers eclipsed by other uses.

There is a responsibility that most probably falls on the shoulder of subspecialized funding agencies and supporting communities to augment research in this area which will continue to benefit patients with drug-resistant epilepsy, in the foreseen future, until, researchers identify less invasive, and more preemptive and efficient methods to treat epilepsy in the future.

In summary, icEEG data is an ideal medium for applications of artificial intelligence and machine learning in real-time. The applications within the domain of epilepsy surgery and seizure localization have lagged behind, however, the transformation is inevitable. Investment from funding agencies is needed to help revamping of care in this sub-group of general population.

Invasive electrophysiology presents some caveats though it remains the standard of care in subset of cases with drug-resistant epilepsy; firstly, the spatial resolution is at the level of local field potential, i.e., in the order of hundreds of millions of neurons and is inherently influenced by the clinical hypothesis and expertise, secondly analysis often performed group-wise not patient-wise and render networks not always outcome-validated clinically meaningful data. That is in addition to the risks presented above. In the long term, and besides advances on this front, it would be desired to continue to investigate new mechanisms of action for pharmacological control of seizures, as well as investigating interventions that prevent epilepsy altogether. Reliable identification of pathologic brain regions is also of interest, functional imaging on the other hand presents unique advantages especially in regard to the spatial resolution, non-invasiveness and safety profile. However, as a general rule non-of the available techniques is a match to the superiority of EEG excellent temporal resolution and are all, to our knowledge, with the exception of MRI imaging – considered complimentary in the presurgical evaluation and do not replace icEEG when the latter is indicated on a clinical basis. Direct cortical brain cooling may prove beneficial in studying the effect of isolating brain regions. Optogenetic approaches present excellent potential for localization of function and dysfunction in epilepsy and modulation of epileptic networks via open- or close-loop circuits if optimized for use in humans – as it enables highly specific and high-resolution activation or deactivation of brain regions/cluster of cells that is induced by light of specific wavelength via light-sensitive genetically modified neuronal receptors and channels (opsins) (Zhao et al., 2015). Genetically encoded voltage gated channels if successfully translated into humans may further improve our ability to map seizure events. These approaches have been successfully applied experimentally in rodents. Several important challenges presently exist: significant progress is still needed in the technical scalability of the approach, safe and effective opsin gene delivery, reliable light delivery in clinical settings, and specific cluster activation *in vivo*. In summary, and while promising, much remains to be understood before application of optogenetics in humans.

## Author Contributions

The author confirms being the sole contributor of this work and has approved it for publication.

## Conflict of Interest Statement

The author declares that the research was conducted in the absence of any commercial or financial relationships that could be construed as a potential conflict of interest.
